# Concomitant active inflammation of myocardium and thyroid, incidental finding in COVID‐19 pandemic: A case report

**DOI:** 10.1002/ccr3.4998

**Published:** 2021-10-28

**Authors:** Golnaz Houshmand, Seyyed Mojtaba Ghorashi, Fatemeh Mirrazeghi, Negar Omidi

**Affiliations:** ^1^ Medical and Research Center Iran University of Medical Sciences Tehran Iran; ^2^ Cardiology Department Tehran Heart Center Tehran University of Medical Sciences Tehran Iran; ^3^ Gilan University of Medical Sciences Rasht Iran

**Keywords:** cardiac magnetic resonance, case report, COVID‐19, myocarditis, SARS‐CoV‐2, Thyroid

## Abstract

SARS‐CoV‐2 could affect every organ either directly or indirectly. We describe a young, healthy man diagnosed with COVID‐19 whose inaugural presentation was concurrent myocarditis and incidental thyroiditis in cardiac magnetic resonance imaging within two months post recovery. This case illustrates the ongoing pro‐inflammatory process several months’ post‐COVID‐19 recovery.

## Graphical abstract

The systemic inflammatory process could be ongoing in patients recovered from COVID‐19 and with mild symptoms at presentation. This suggests that the ongoing inflammatory process is independent of severity at presentation and course of COVID‐19.

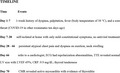

## 1 INTRODUCTION

**FIGURE 1:**
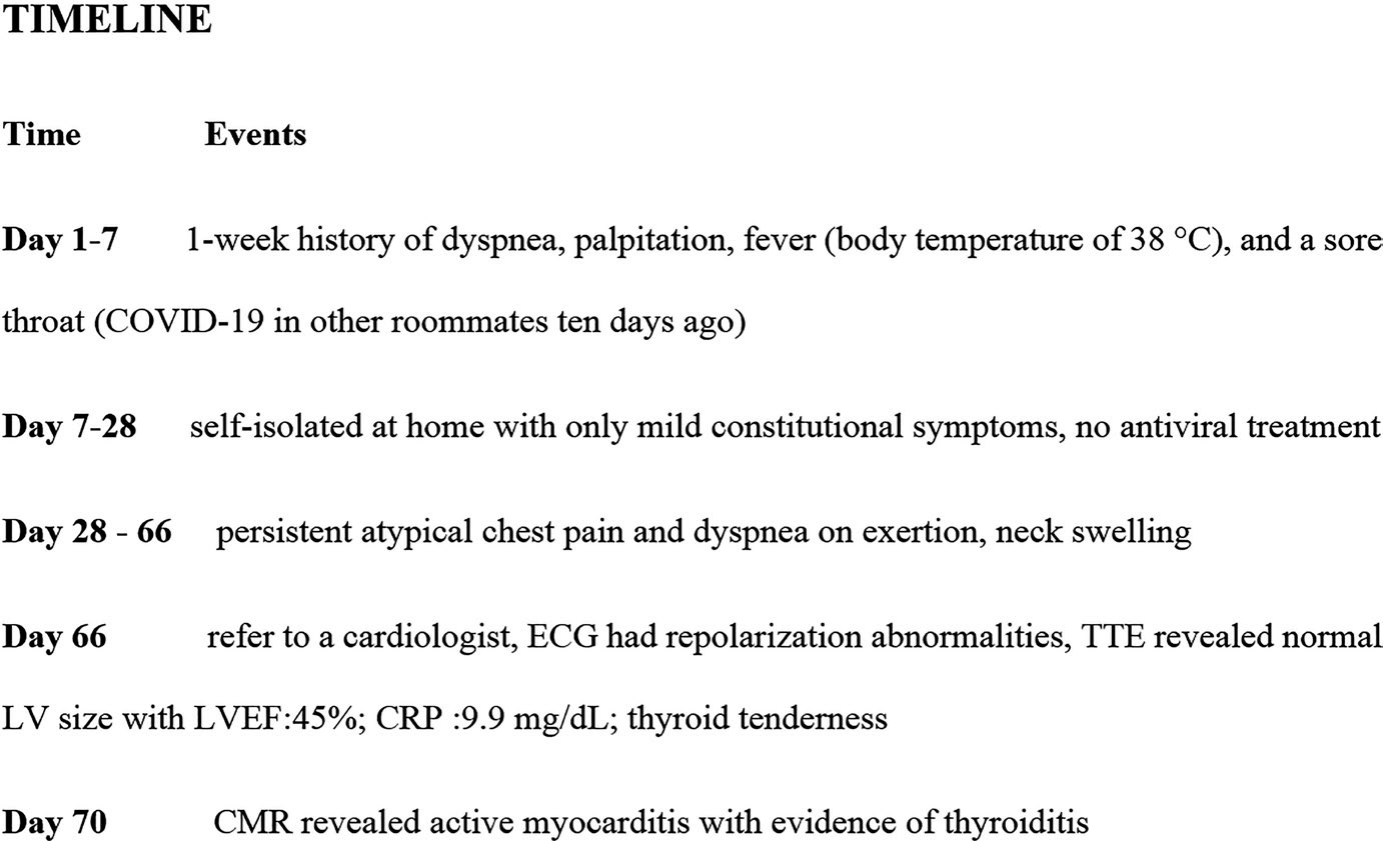
Timeline diagram outlining patient's clinical course

Coronavirus 2019 Disease (COVID‐19) could impact all organs directly or indirectly. COVID‐19‐induced myocardial injury has an incidence of somewhere between 7% to 23% and is also associated with increased mortality.^1^, ^2^ We herein describe a young man with a history of COVID‐19 infection, who was referred for evaluation of chest pain and left ventricular (LV) systolic dysfunction with an incidental finding of thyroiditis around two months of post‐recovery. To the best of our knowledge, there are no papers published so far on concomitant myocarditis and thyroiditis, several months after the recovery of COVID‐19. This case highlights the fact that a systematic pro‐inflammatory process would continue months after the COVID‐19 infection. Figure 1 illustrates the timeline diagram outlining patient's clinical course. The report conformed to the principles of Helsinki.

### 1.1 CASE PRESENTATION

**FIGURE 2:**
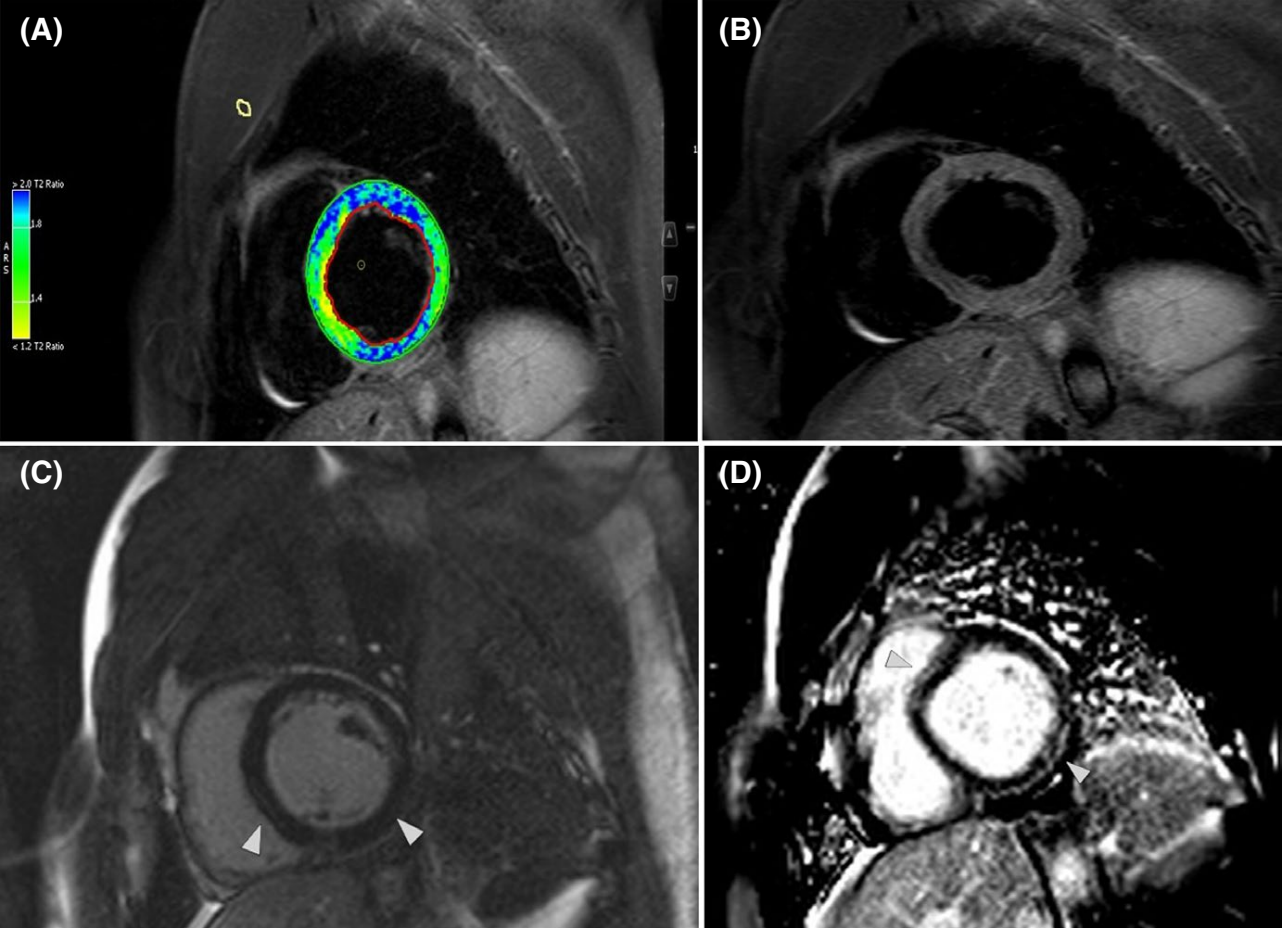
STIR and late gadolinium enhancement sequences (LGE). (A) STIR short axis sequence revealed increased signal intensity and edema in the basal to midpart of septum and lateral walls. (B) LGE sequence revealed subepicardial and mid‐wall enhancement in the basal to midpart of the septum and lateral walls

**FIGURE 3:**
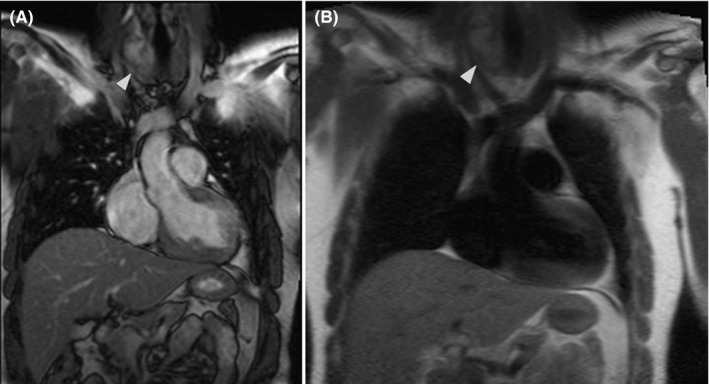
Steady‐state free precession (SSFP) sequence revealed irregular and large right thyroid lobe with cystic components

A 25‐year‐old man with a 1‐week history of dyspnea, palpitation, fever (body temperature of 38°C), and a sore throat was diagnosed with COVID‐19 infection in August 2020. The diagnosis was made based on the clinical symptoms; as other roommates were also diagnosed with COVID‐19 based on real‐time polymerase chain reaction (RT‐PCR). His past medical and family history were unremarkable. He self‐isolated himself and did not admitted to the hospital or received any antiviral medications and recovered from COVID‐19 in two weeks. Since then, the patient had a complaint of persistent atypical chest pain and dyspnea on exertion and was referred to the cardiologist in November 2020, he also mentioned ongoing sore throat. The electrocardiogram demonstrated a nonspecific change in repolarization, and the transthoracic echocardiography revealed normal left ventricular size with a left ventricular ejection fraction (LVEF) of 45%. Laboratory examinations demonstrated a C‐reactive protein (CRP) level of 9.9 mg/dl (normal range<0.5 mg/dl), white blood cell count of 7100/μl, with a lymphocyte percentage of 18.8% and high troponin I level. The cardiac magnetic resonance imaging (CMR) depicted mildly increased LV‐indexed volumes with moderately reduced ejection fraction (LVEF of 44%, LV end‐diastolic volume index of 101 ml/m^2^, global longitudinal strain of −12.5%, and global circumferential strain of −16.01%). The right ventricular indexed volumes and ejection fraction were normal (RVEF:51% and RV end‐diastolic volume index: 95 ml/m^2^). The short tau inversion recovery sequences (STIR) revealed an increased signal intensity in the basal to mid part of the septum and lateral walls. The late gadolinium enhancement (LGE) images showed a subepicardial and mid‐wall enhancement in the basal to midpart of the septum and lateral walls (Figure 2). There was mild bilateral pleural effusion with evidence of pleural‐based infiltration in the left lower lung. The CMR finding is in keeping with active myocarditis. The incidental extra‐cardiac finding in anatomic images was irregular and large right thyroid lobe with fluid containing lesions and heterogeneous signal intensity (Figure 3) which was confirmed by thyroid sonography. The thyroid function test revealed a thyroxin (T4) level of 9.90 mcg/dl (normal range: 4.2–12.5 mcg/dl), triiodothyronine (T3) of 2.23 nmol/L (normal range: 1.10–2.90 nmol/L), and thyroid‐stimulating hormone (TSH) of 1.3 mIU/ml (normal range: 0.3–5.5 mIU/ml). According to the clinical finding of neck swelling and thyroid tenderness in the physical examination, the diagnosis of concomitant subacute thyroiditis and active myocarditis was considered, and treatment with a beta‐blocker, angiotensin‐converting enzyme inhibitor, and anti‐inflammatory agent were started, and follow‐up was planned.

## 2 DISCUSSION

In the present case, we addressed the COVID‐19‐related delayed systemic inflammatory reaction in both the myocardium and thyroid in a young man after an episode of COVID‐19 with unremarkable initial symptoms. The diagnostic workup showed concurrent active myocarditis with thyroiditis within months after recovery.

COVID‐19 infection, even mild, promotes a pro‐inflammatory state such as inducing oxidative stress and cytokine surge as evident by increased plasma interleukin, CRP, and monocyte chemoattractant protein‐1 (MCP‐1) which could mediate acute myocardial injury even after recovery.^3^, ^4^ According to previous studies, a high burden of inflammation is associated with cardiovascular involvement. The mechanism of myocarditis is considered to be immune‐mediated indirect myocardial inflammation due to inflammatory cytokines or direct viral inclusion.^1^, ^5^ The reported frequency of CMR‐documented cardiac involvement in patients with ongoing symptoms was 58%.^6^ Puntmann and Rajpal studies also showed a high frequency of persistent inflammation in patients with recovered COVID‐19 which were independent of severity at presentation.^7^, ^8^, ^9^, ^10^, ^11^, ^12^, ^13^

The frequency of thyroid dysfunction in patients with mild to moderate COVID‐19 is estimated to be 13.2%. Reports of COVID‐19‐induced thyroiditis suggest either a direct or indirect effect on thyroid function, with low T3 and serum TSH levels appear to have prognostic significance.^6^, ^7^, ^8^, ^14^, ^15^

The presentation of thyroid disorder in COVID‐19 could be hypothyroidism, thyrotoxicosis, and nonthyroidal illness syndrome. Also, subacute thyroiditis could occur in the post‐recovery phase.^8^ The underlying mechanism for decreased TSH level in the acute phase of severe acute respiratory distress syndrome (SARS) could be central hypothyroidism due to reversible hypophysitis or hypothalamic effect as an adaptation to a severe illness in intensive care unit (ICU) setting.^5^, ^16^, ^17^, ^18^ According to reports, hypothyroidism could suggest a possible mechanism of symptoms in the post‐COVID‐19 recovery phase.^14^, ^15^, ^18^ We assumed that COVID‐19 patients could be prone to either virus or immune‐mediated thyroid damage with the same mechanisms of myocardial involvement resulting in the development of thyroiditis.

Since post‐COVID‐19 thyroid disorder would affect general patients’ condition and myocardium, struggling with complicated cases is crucial to keep the probable sequels in mind.

## 3 CONCLUSION

Patients recovered from COVID‐19 may suffer from a wide range of symptoms and also may have a constant inflammatory state and late sequels in different organs. This case suggests that one should be aware of the presence of we presented delayed thyroiditis and myocarditis in a recovery phase. The World Health Organization had not recommended the assessment of thyroid function for COVID‐19. The diagnosis of thyroiditis in the post‐COVID‐19 stage could be difficult considering the nonspecific symptoms. However, timely recognition is critical. There remains poor insight into the extent of the pro‐inflammatory process in COVID‐19 patients; a cohort study of patients with myocardial and thyroid inflammation could be of value.

## CONFLICT OF INTEREST

Nothing has been declared.

## AUTHOR CONTRIBUTIONS

NO and GH contributed to the conception of the work MGH and FM contributed to data acquisition. All authors have approved the submitted version.

## ETHICAL APPROVAL

The project was approved by the Medical Ethics Committee of Rajaei Cardiovascular Research Center. The authors confirm that the report conformed to the principles of Helsinki.

## 4 CONSENT

The informed consent for publication was obtained from the patient.

## Data Availability

The data of this article will be shared on request.
